# PINK1 Protects Against Gentamicin-Induced Sensory Hair Cell Damage: Possible Relation to Induction of Autophagy and Inhibition of p53 Signal Pathway

**DOI:** 10.3389/fnmol.2018.00403

**Published:** 2018-11-12

**Authors:** Qianqian Yang, Yiwei Zhou, Haiyan Yin, Hongrui Li, Meijuan Zhou, Gaoying Sun, Zhixin Cao, Rongjun Man, Haibo Wang, Jianfeng Li

**Affiliations:** ^1^Otolaryngology-Head and Neck Surgery, Shandong Provincial Hospital Affiliated to Shandong University, Jinan, China; ^2^Department of Pathology and Pathophysiology, School of Medicine, Shandong University, Jinan, China; ^3^Weifang Nursing Vocational College, Weifang, China; ^4^Shandong Provincial Key Laboratory of Otology, Jinan, China; ^5^Department of Pathology, Shandong Provincial Hospital Affiliated to Shandong University, Jinan, China; ^6^Department of Otolaryngology Head and Neck Surgery, Zibo Central Hospital, Zibo, China

**Keywords:** phosphatase and tensin homolog (PTEN)-induced putative kinase 1 (PINK1), gentamicin (GM), ROS, autophagy, p53

## Abstract

Phosphatase and tensin homolog (PTEN)-induced putative kinase 1 (PINK1) is a gatekeeper of mitochondrial quality control. The present study was aimed to examine whether PINK1 possesses a protective function against gentamicin (GM)-induced sensory hair cell (HC) damage *in vitro*. The formation of parkin particles (a marker revealing the activation of PINK1 pathway which is a substrate of PINK1 and could signal depolarized mitochondria for clearance) and autophagy were determined by immunofluorescence staining. The expressions of PINK1, LC3B, cleaved-caspase 3 and p53 were measured by Western blotting. The levels of reactive oxygen species (ROS) and apoptosis were respectively evaluated by DCFH-DA staining, Annexin V Apoptosis Detection Kit and TUNEL staining. Cell viability was tested by a CCK8 kit. We found that treatment of 400 μM GM elicited the formation of ROS, which, in turn, led to PINK1 degradation, parkin recruitment, autophagy formation, an increase of p53 and cleaved-caspase 3 in HEI-OC1 cells and murine HCs. In contrast, co-treatment with ROS scavenger N-acetyl-L-cysteine (NAC) inhibited parkin recruitment, alleviated autophagy and p53 pathway-related damaged-cell elimination. Moreover, PINK1 interference contributed to a decrease of autophagy but an increase of p53 level in HEI-OC1 cells in response to GM stimulus. Findings from this work indicate that PINK1 alleviates the GM-elicited ototoxicity via induction of autophagy and resistance the increase of p53 in HCs.

## Introduction

Phosphatase and tensin homolog (PTEN)-induced putative kinase 1 (PINK1) is a 581-aa polypeptide with a functional serine/threonine kinase domain and a N-terminal mitochondrial targeting signal (MTS; Zhou et al., 2008), which is highly expressed in tissues with high energy need (Pickrell and Youle, 2015), including sensory hair cells (HCs) of inner ear cochlea described in our previous study (Yang et al., 2018). Mounting evidence shows that PINK1 is a gatekeeper of mitochondrial quality control in addition to its critical regulatory function in several cellular processes through generation of mitochondrial-derived vesicle, mediation of mitophagy, regulation of calcium efflux from mitochondria, phosphorylation of targeting proteins, and so forth. (Leites and Morais, 2018). PINK1 globally accumulates on depolarized mitochondria in response to uncoupler carbonyl cyanide m-chlorophenyl hydrazine (CCCP) treatment, which, in turn, leads to the accumulation of reactive oxygen species (ROS) and activates PINK1’s downstream E3 ubiquitin ligase, parkin, by PINK1 phosphorylation, leading to the aggregation of parkin from cytoplasm to damaged mitochondria and, ultimately, clearing the entire organelles in a manner depending on autophago-lysosome pathway, a process called mitophagy (Matsuda et al., 2010; Sun et al., 2012; McLelland et al., 2014; Sánchez-Rodríguez et al., 2016). As a major source of ROS, the dysfunction of mitochondria results in overproduction of ROS, which would elicit oxidation of important mitochondrial proteins, lipids and DNA, exacerbating mitochondrial damage. The impaired mitochondria consequently result in mitophagy through the interaction of microtubule-associated protein light chain 3 (LC3) and other functional proteins at outer mitochondrial membrane (Youle and Narendra, 2011; Prieto-Domínguez et al., 2016). It is now convincingly confirmed that autophagy is a critical quality control way of cells to degrade damaged organelles or cellular components by the combination of LC3 coated phagophores and lysosomes to ensure normal cell function as well as to help damaged cells survive under various stresses, including the administration of certain ototoxic drugs (Stolz et al., 2014).

Aminoglycosides were widely used in clinical treatment for its low cost and broad antibacterial spectrum against the majority of gram-negative bacteria (Durante-Mangoni et al., 2009). However, their therapy is compromised by serious side-effects, in particular, the ototoxicity, leading to even permanent sensorineural hearing damage, which significantly lowers the life quality of patients and limits their use in clinic (Baguley et al., 2013; Lanvers-Kaminsky et al., 2017). Aminoglycosides can cause HC damage from the outer hair cells (OHCs) of the basal turn, followed by the transfer to upper turns and inner hair cells (IHCs) with continue drug exposure (Xie et al., 2011). As a member of aminoglycosides, gentamicin (GM) could dissipate mitochondrial membrane potentials of cochlear HCs and induce oxidative stress (Jiang et al., 2016). Substantial evidence shows that the overproduced ROS could trigger high level of autophagy and apoptosis of HC (Yang et al., 2011; Yuan et al., 2015). Though aminoglycosides have been used for a long period of time, exact mechanisms involved in their damage effects have still not been completely clear, thereby seeking for the protective mechanism so as to efficiently reduce their side-effects is imperative.

As a tumor suppressor, p53 regulates a wide range of cellular metabolism, cell cycle, the development of tumors and maintains cellular homeostasis under stresses, which has long been recognized as an important cell death mediator (Maddocks and Vousden, 2011; Wang et al., 2017). p53 could mediate apoptosis by transactivating proapoptotic genes and directly interacting with Bax or other Bcl2 family members, activating caspase cascade, preventing the accumulation of abnormal cells (Crighton and Ryan, 2004). Studies show that GM could provoke ROS generation and intrinsic apoptosis by disrupting mitochondrial membrane (Servais et al., 2005; Denamur et al., 2016). Moreover, p53 also plays an important role in GM-induced cochlear HC death through mitochondrial-associated apoptosis (Warchol, 2010; Coffin et al., 2013), and the accumulation of p53 is an early sign of apoptotic change in the aging HCs (Xu et al., 2017).

It has been documented that the deficiency of PINK1 is related to a broad spectrum of diseases (Mizumura et al., 2014; Agnihotri et al., 2017; Eid and Kondo, 2017; Rovira-Llopis et al., 2017; Zhou et al., 2017), highlighting the significance of PINK1 pathway as a pivotal mechanism responsible for the maintenance of human healthy. However, whether PINK1 pathway has function in GM-generated HC damage or not has not been researched as yet. Therefore, the present study was designed to examine the possible correlation between PINK1 and GM-induced sensory HC damage *in vitro*, with special attention given to p53 activities in this process.

## Materials and Methods

### Cell Culture and Cell Line

HEI-OC1 cells, a cell line possessed HC-like properties, which was derived from murine organ of Corti, were cultured in high-glucose DMEM (Gibco BRL, Grand Island, NY, USA) mixed with 10% volume of fetal bovine serum (Gibco BRL, Grand Island, NY, USA) without antibiotics in incubators of 5% CO_2_ and at 33°C.

### Basal Membrane Culture

P4 C57bl/6 murine cochleae were carefully taken out. The stria vascularis, spiral ligament, Reissner’s and tectorial membrane were carefully cut away, leaving the basal membrane stuck to a glass of coverslip by use of Cell-Tak (BD Biosciences, Franklin Lakes, NJ, USA). The explants were treated with drugs for 3 h, 6 h, 12 h and 24 h, and then, subjected to immunofluorescence staining and Western blotting analysis. This study and the protocol were carried out in accordance with the Animal Care Committee of Shandong University, Jinan, China (No. ECAESDUSM 20123011).

### Drug Treatment

The cells or cochlear explants were exposed to 400 μM GM (G8170, Solarbio) or exposed to 400 μM GM together with 2 mM N-acetyl-L-cysteine (NAC; Sigma-Aldrich) after pretreated by 2 mM NAC for 2 h. Then they were taken for different measurements at 1 h, 3 h, 6 h, 12 h and 24 h. The inhibitors of autophagy and p53 pathway, 5 mM 3-methyladenine (3-MA; M9281, Sigma-Aldrich) and 10 μM Pifithrin-α (PFTα; S2929, Selleck Chemicals) respectively, were used on the basis of interference of PINK1 expression in HEI-OC1 cells.

### ROS Detection

The intracellular ROS level was detected by use of DCFH-DA staining (D6883, Sigma Technologies). Cells after treatment were washed by pre-warmed PBS, then stained by 10 μM DCFH-DA in serum free DMEM for 30 min. Fluorescent intensity level was measured through fluorescent microscopy and FACS Calibur system (BD Biosciences). The cell counter tool in ImageJ software was used for HEI-OC1 cells counting. The ratio of ROS positive cells meant DCFH-DA positive cells/total HEI-OC1 cells in the same view.

### Protein Extraction and Western Blotting

The total protein of different treated cells or tissues were extracted by RIPA lysis buffer (P0013B, Beyotime Institute of Biotechnology) and centrifugation. After the concentration measurements by BCA assay kit (Shenergy Biocolor Bioscience and Technology Company, Shanghai, China), equal amount of protein was denatured and separated by 12% SDS-PAGE electrophoresis, then transferred to polyvinylidene fluoride membranes (PVDF; Immobilon-P, Cat. No. IPVH00010). The membranes were blocked in TBS containing 0.05% tween20 (TBST) with 5% BSA and incubated with related primary antibodies in TBST with 3% BSA overnight. Then, they were incubated in second antibodies for 1 h, detected by use of the ECL kit (Millipore, Hercules, CA, USA) and analyzed by ImageJ software. The primary antibodies were used as follows: anti-PINK1 antibody (ab75487, Abcam), anti-p53 antibody (sc1312, Santa Cruz), anti-LC3B antibody (3868s, CST) and anti-cleaved caspase 3 antibody (9664s, CST).

### PINK1 Interference

A PINK1-mus specific 21-nucleotide siRNA was designed by GenePharma (Shanghai, China) to silence the expression of PINK1 in HEI-OC1 cells. Nine microliter Lipofectamine 3000™ (Invitrogen, Carlsbad, CA, USA) and 140 pmol siRNA duplexes were added to appropriate volume of Opti-MEM (Gibco BRL, Grand Island, NY, USA) to make a 2-ml working solution. The sequences of PINK1-siRNA and the negative control (NC) group were listed at the bottom in Table 1. The efficiency of interference was measured by Western blotting. After the interference for 24 h, 400 μM GM, or the co-treatment of GM and autophagy inhibitor 3-MA or p53 inhibitor PFTα, were given after the pretreatment of the inhibitors for 2 h. After drug treatments, cells were harvested for assays of mitochondrial membrane potential, parkin particle formation, autophagy, cell viability and apoptosis.

**Table 1:** SiRNA duplexes used in this study.

Name	Sense sequence (5’ to 3’)	Antisense sequence (5’ to 3’)
PINK1-siRNA	GCACACUGUUCCUCGUUAUTT	AUAACGAGGAACAGUGUGCTT
Negative control	UUCUCCGAACGUGUCACGUTT	ACGUGACACGUUCGGAGAATT

### Immunofluorescence

Cells were stained with 200 nM Mitotracker-deep Red FM (M22426, Life Technologies) in serum-free DMEM at 37°C absent from light for 30 min, then fixation with 4% PFA for hours. The fixed basal membrane and cells were then permeabilized for 30 min with 1% Triton X-100 or 0.2% Triton X-100 respectively, blocked with 1% BSA in PBS for 1 h and incubated with the primary antibodies overnight. The primary antibodies used in our present study were anti-PINK1 antibody (ab75487, Abcam), anti-Myosin VIIa antibody (25-6790, Proteus-biosciences; 138-1, DSHB), anti-parkin antibody (ab15954, Abcam), anti-Tom 20 antibody (sc-11415, Santa Cruz), anti-Lamp1 antibody (sc-8098, Santa Cruz), anti-LC3B antibody (3868s, CST), anti-p53 antibody (sc1312, Santa Cruz) and anti-cleaved caspase 3 antibody (9664s, CST). Then, they were incubated with secondary fluorescent antibodies and DAPI (D9542, Sigma-Aldrich) for 1 h in dark after three times wash by PBS. The specimens then were observed through a laser scanning confocal microscope (TSC SPE, LEICA).

### Cell Viability Measurement

Cells were seeded at the density of 3,000 cells/well in a 96-well plate and allowed to attach overnight, then was treated by PINK1-siRNA or NC duplexes. Twenty-four hours later, they were incubated with 400 μM GM, or GM and autophagy inhibitor or p53 inhibitor for another 24 h after the preconditioning of inhibitors for 2 h correspondingly. Then 10 μl per well CCK-8 reagent (96992, Sigma-Aldrich) was added in incubation for 2 h. Absorbance at 450 nm was detected by ELISA reader (Multiskan MK3) for cell viability and the optical density (OD) of NC group was taken as 100% of viability. Cell viability was calculated according to the following formula: cell viability(%) = OD_experiment_/OD_control_ × 100% (OD_blank_ as used to zero).

### TUNEL Staining

Click-iT^®^ Plus TUNEL Assay (Life technologies, Washington, DC, USA) was used to detect the apoptotic level of auditory HCs *in vitro* following its protocol, which can be followed by other stainings such as DAPI and other needed antibodies. The results were detected by a Leica confocal laser scanning microscopy.

### Apoptosis Analyzed by Flow Cytometry

Cell apoptosis were also measured by use of a FITC Annexin V Apoptosis Detection Kit (556547, BD Pharmingen™, RUO). Cells were harvested and washed twice with cold PBS solution, and resuspended with 100 μl 1× binding buffer softly. Five microliter Annexin V and 5 μl propidium iodide (PI) were added to each group and incubated in dark room for 15 min. 20,000 cells of each group were measured by a FACS Calibur system (BD Biosciences) and then evaluated with the FlowJo 7.6 software.

### Mitochondrial Membrane Potential Detection

After different treatments, cells were incubated with 1 μM Rhodamine 123 (C2007, Beyotime Institute of Biotechnology) at 33°C for 30 min shielded from light. Then the florescence density was measured through a laser scanning confocal microscope and FACS Calibur system (BD Biosciences).

### Statistical Analyses

Data were shown as mean ± SEM, and analyzed by one-way analysis of variance (ANOVA) followed by Least-Significant Difference (LSD) *post hoc* test or independent *t*-test. The correlation analysis was made by Pearson’s correlation analysis. All the experiments were repeated at least three times (*n* ≥ 3). *p* < 0.05 was considered statistically significant. The *p* values were showed in the Supplementary Materials.

## Results

### Treatment by 400 μM GM Could Cause ROS Formation in HEI-OC1 Cells

**Figure 1:**
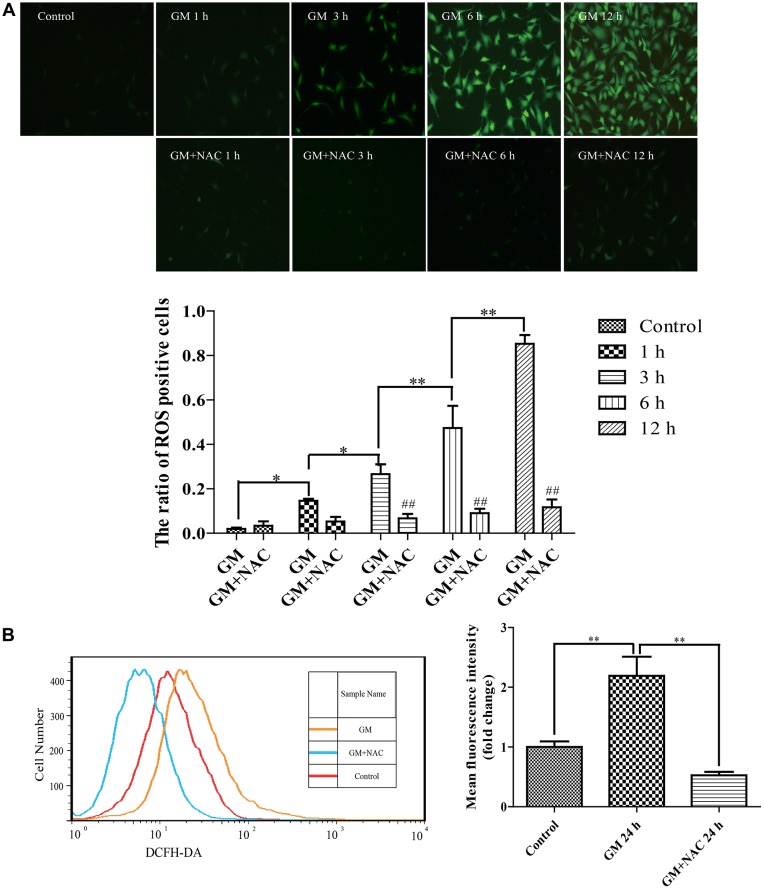
Exposure to 400 μM gentamicin (GM) led to the overproduction of reactive oxygen species (ROS) in HEI-OC1 cells, which could be almost cleared by N-acetyl-L-Cysteine (NAC) co-treatment. **(A)** Green fluorescence positive cells (DCFH-DA positive cells) represented cells with ROS formation. GM could make the intracellular formation of ROS, and the number of DCFH-DA positive cells increased as time went by, indicating that GM could make a time-dependent ROS production. As a ROS scavenger, NAC could effectively reduce the level of ROS induced by GM, especially at 3 h, 6 h and 12 h. ^##^*p* < 0.01 vs. corresponding GM groups, *p < 0.05, **p < 0.01. **(B)** The fluorescence intensity of DCFH-DA staining was measured by flow cytometry, **p < 0.01. Results showed that GM treatment for 24 h could increase the fluorescence intensity of DCFH-DA in HEI-OC1 cells. NAC co-treatment could obviously decrease the intensity of DCFH-DA induced by GM. The cells of control group were seeded and measured together with other groups.

DCFH-DA fluorescence staining showed that 400 μM GM exposure caused ROS generation in HEI-OC1 cells and the level of which increased as time went by, indicating that GM stimulus induced ROS production in a time-dependent pattern. The co-treatment of 2 mM ROS scavenger, NAC, could effectively reduce the production of ROS induced by GM (Figure 1A). The fluorescence intensity of DCFH-DA after 24 h of stimulus was also detected by flow cytometry. Results revealed that NAC co-treatment could effectively inhibit the production of ROS induced by GM (Figure 1B).

### The Expression of PINK1 in HEI-OC1 Cells Was Influenced by GM Exposure

**Figure 2:**
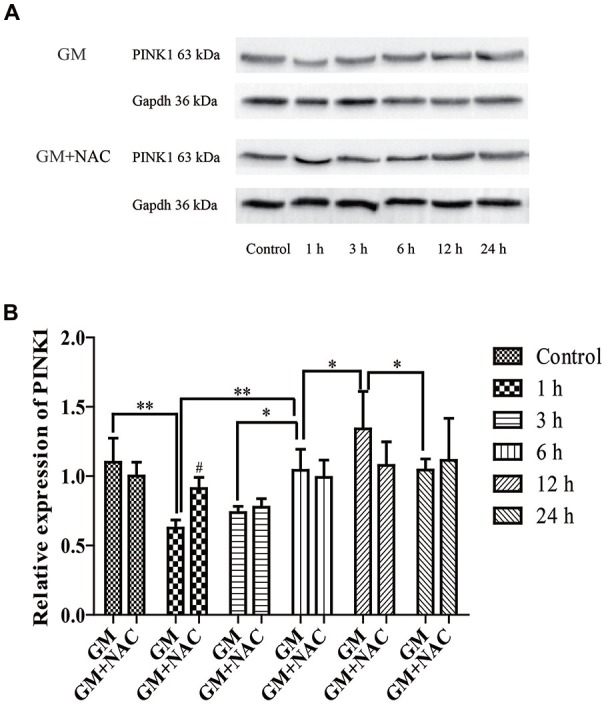
The expression of phosphatase and tensin homolog (PTEN)-induced putative kinase 1 (PINK1) in HEI-OC1 cells was influenced by GM stimulus. **(A)** The expression of PINK1 in response to GM and GM plus NAC co-treatment were measured by use of Western blotting. **(B)** Analysis of gray-degree showed that the expression of PINK1 decreased immediately after GM treatment for 1 h, then increased step by step reaching the peak at 12 h followed by a decline at 24 h. The co-treatment of NAC could inhibit the changes of PINK1 expression induced by GM, as the changes of NAC co-treated groups showed no statistic difference in contrast to control group. NAC could effectively inhibit the decrease of PINK1 expression, especially at early period (1 h), ^#^*p* < 0.05 vs. GM group at 1 h, *p < 0.05, **p < 0.01.

The expression of PINK1 in HEI-OC1 cells showed a sharp decline at 1 h, then increased step by step reaching the peak at 12 h, followed by a decrease again at 24 h in response to a stimulus of GM. PINK1 in GM and NAC co-treatment groups showed no statistic difference vs. control group, implying that the changes of PINK1 expression in GM groups were strongly suppressed by co-treatment of NAC, especially at the early time point (1 h; Figure 2).

### GM Could Cause Parkin Recruitment to Mitochondria Which Was Reduced by Co-treatment of NAC

**Figure 3:**
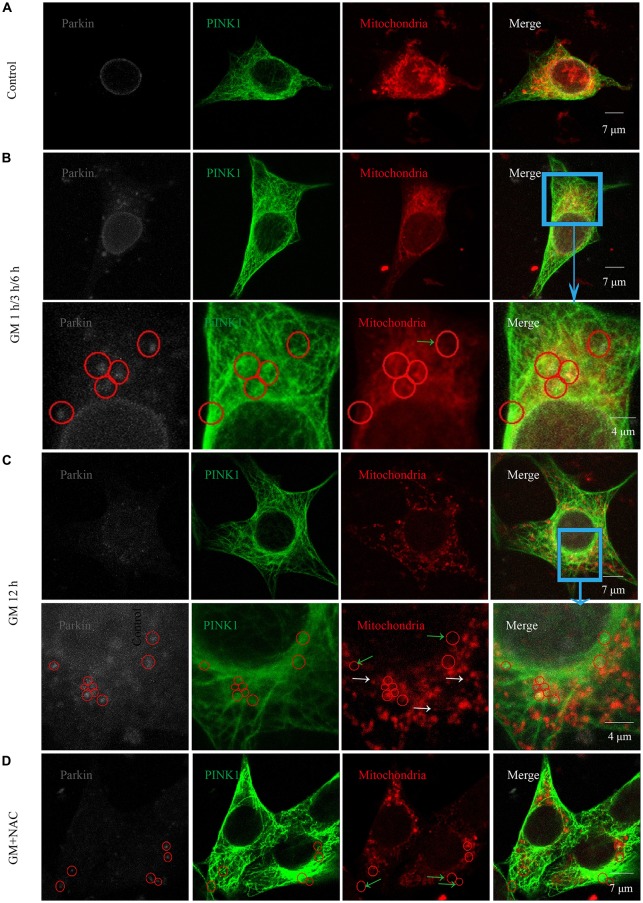
GM treatment led to the aggregation of parkin to mitochondria which was reduced by co-treatment of NAC. **(A)** In the experiment, parkin marked by gray fluorescence, PINK1 by green fluorescence and mitochondria by red fluorescence. There were almost no parkin particles in cytoplasm of control HEI-OC1 cells. **(B)** Obvious parkin particles were detected colocalized with PINK1 and mitochondria (red circles) after GM treatment for 1 h, 3 h and 6 h, and there were also some parkin+/PINK1+ double positive particles not colocalize with mitochondria (green arrows). The major difference of the designed time points was the ratio of parkin-particle positive cells, which would be showed in Figure 4. **(C)** In addition to the parkin particles, including the ones not in mitochondria (green arrows), cells in the group treated for 12 h showed a large number of punctiform mitochondria (white arrows). **(D)** Cells of GM and NAC co-treatment groups at all the time points showed sharply decreased number of parkin particles distributed in the cytoplasm.

**Figure 4:**
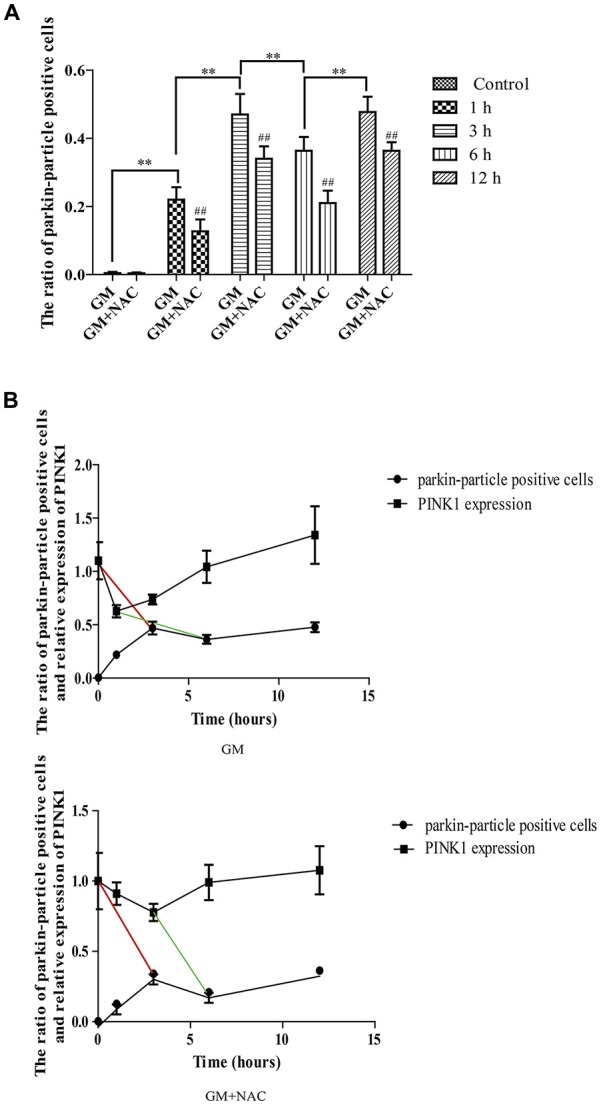
The ratio of parkin-particle positive cells changed following the function of PINK1. **(A)** The ratio of parkin-particle positive cells increased significantly in response to GM exposure for 1 h compared with control group which had almost no parkin particles. The ratio risen to the peak at 3 h then lessened at 6 h, followed by an increase again at 12 h. NAC co-treatment could alleviated the changes of the ratio of parkin-particle positive cells treated by GM. ^##^*p* < 0.01 vs. corresponding GM groups, **p < 0.01, results were shown as mean ± SEM. **(B)** We also compared the changes of PINK1 expression and the ratio of parkin-particle positive cells after GM stimulus and NAC co-treatment. Results showed the delayed peaks (red line segments) and valleys (green line segments) of parkin-particle positive cells in contrast with PINK1 expression. NAC co-treatment could mitigate the changes of both PINK1 expression and the ratio of parkin-particle positive cells after GM exposure.

Immunofluorescence showed the colocalization of parkin, PINK1 and mitochondria after GM treatment at 1 h, 3 h, 6 h and 12 h (Figures 3B,C, red circles), including some particles in close proximity to but not overlapped with mitochondria (Figures 3B,C, green arrows), in comparison with control group, which showed almost no parkin particles in cytoplasm (Figure 3A). What’s more, there were many punctiform mitochondria at 12 h after GM treatment (Figure 3C, white arrows). The parkin particles gathered to mitochondria were effectively reduced by NAC co-treatment (Figure 3D). All the data above indicated that GM could cause serious mitochondrial damage, activate PINK1/parkin pathway and recruit parkin to injured mitochondria, which was tightly related to the intracellular ROS induced by GM. We had also counted the number of parkin-particle positive cells of different groups. Results revealed that GM led to the formation of parkin particles in HEI-OC1 cells immediately after treatment. And the ratio went up, reaching peak at 3 h, then reduced at 6 h followed by a rise again at 12 h, the changes of which was alleviated by use of NAC (Figure 4A). In addition, we compared the changes of PINK1 expression and the ratio of parkin-particle positive cells in response to GM stimulus and NAC co-treatment. We found that the ratio of parkin-particle positive cells changed following the variation of PINK1 expression, the peaks (Figure 4B, red broken lines) and valleys (Figure 4B, green broken lines) appeared hours later, which could be mitigated by NAC co-stimulus.

## GM Treatment Caused Severe Mitochondrial Damage and an Increase of p53 in HEI-OC1 Cells, Both of Which Could be Partially Alleviated by Use of NAC

**Figure 5:**
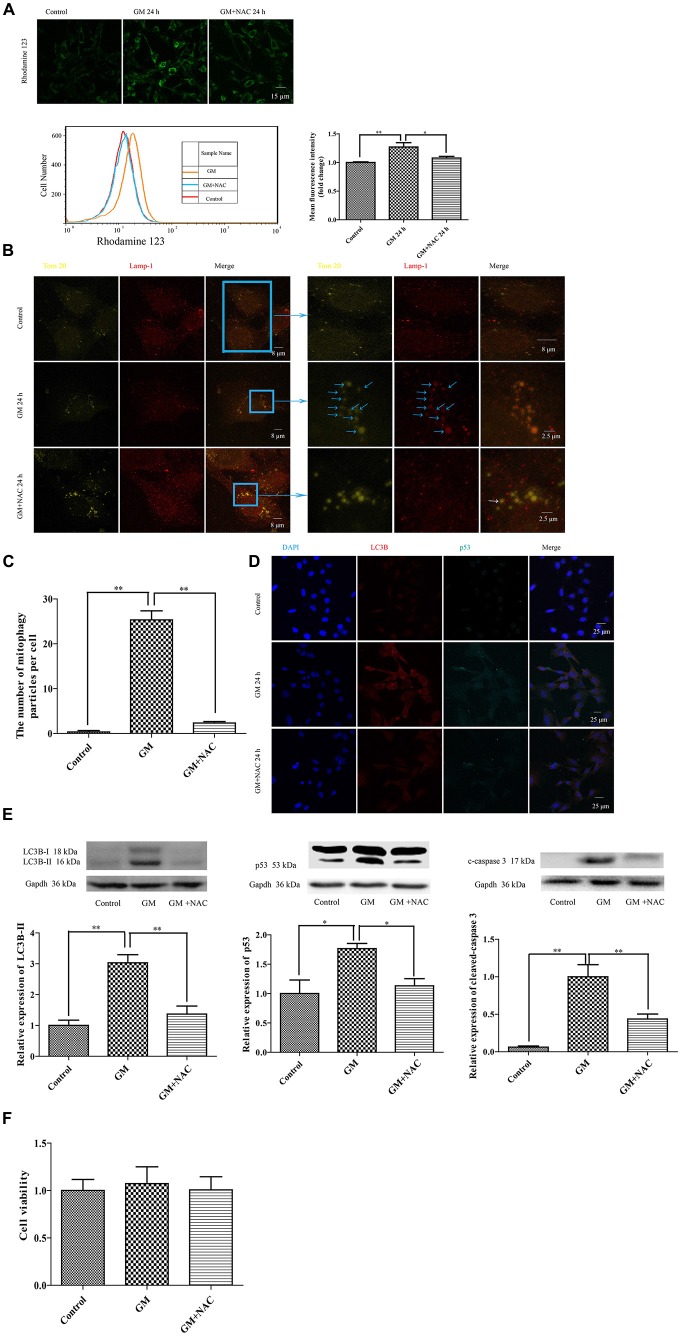
GM treatment caused mitochondrial membrane potential damage, severe mitophagy and an increase of p53 in HEI-OC1, which could be reduced by co-treatment of NAC. **(A)** Mitochondrial membrane potential was marked by use of the sensitive probe Rhodamine 123, and detected through the measurement of fluorescence intensity. GM stimulated cells showed an increase of Rhodamine 123 fluorescence at 24 h, which could be reduced by use of NAC. *p < 0.05, **p < 0.01. **(B)** Mitochondria and lysosomes were labeled by Tom 20 (yellow-green fluorescence) and Lamp-1 (red fluorescence) respectively. Immunofluorescence staining revealed no colocalization of mitochondria and lysosomes in control group. However, more than 20 colocalized particles of mitochondria and lysosomes were observed around the nuclei 24 h after GM treatment, the color of which were orange because of the complete overlap of yellow-green and red (blue arrows). The co-treatment of GM and NAC led to an effective reduce of the number of mitophagy (white arrow). **(C)** Quantification analysis of mitophagy level revealed the mitophagy particles were induced by GM stimulus and inhibited by NAC co-treatment. **p < 0.01. **(D)** The treatment of GM induced high level of LC3B and p53, which could be cut down by co-treatment of NAC. **(E)** Western blotting analysis verified the results of immunofluorescence, we could see the increase of LC3B-II, p53 and cleaved-caspase 3 after 24 h of GM stimulus, which could be decreased by NAC co-treatment. *p < 0.05, **p < 0.01. **(F)** Cell viability analysis through CCK8 kit showed that there was no obvious difference among control, GM and NAC co-treatment groups.

The mitochondrial membrane potential sensitive probe, Rhodamine 123, was chosen to measure the mitochondrial membrane potential after GM or GM/NAC co-treatment. Results showed the increase of fluorescence intensity after GM treatment, indicating the damage of mitochondrial membrane potential, which could be alleviated by use of NAC (Figure 5A). Tom 20 and Lamp-1 were used to label mitochondria and lysosomes respectively. After GM treatment for 24 h, we definitely detected more than 20 colocalization of mitochondria and lysosomes in the perinuclear region (Figures 5B,C, blue arrows) compared with control group, which implied severe mitophagy caused by GM. The number of autophagosome were effectively reduced by GM plus NAC co-treatment, as there was even no orange particle, forming by the overlapping of yellow-green and red, in NAC co-treatment group (Figures 5B,C, white arrow). We also used LC3B, p53 and cleaved-caspase 3 to measure the degree of autophagy and p53 pathway induced by GM. Results showed that GM could lead to high level of autophagy, aggregation of p53 and activation of caspase 3 in contrast to control group, which could be efficiently cut down by NAC (Figures 5D,E). However, we did not detect obvious cell death in GM-treated cells (Figure 5F). We deduce from the above results that ROS plays an important role in GM-caused autophagy and accumulation of p53 in HEI-OC1 cells.

## PINK1 Could Protect Against GM-Induced Damage by Promoting Autophagy and Inhibiting the Increase of p53 in HEI-OC1 Cells

**Figure 6:**
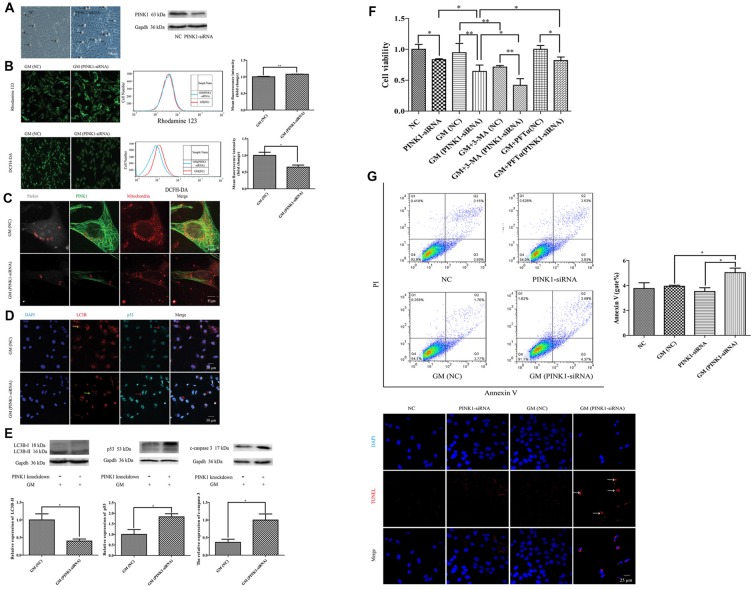
PINK1 could protect HEI-OC1 cells from GM-induced damage by promoting autophagy and inhibiting the excessive activation of p53 pathway. **(A)** There were more morphological abnormal cells in PINK1-silenced group even without GM exposure (black arrows). Western blotting showed the decrease of PINK1 expression in HEI-OC1 treated with specific PINK1-siRNA in comparison with NC group. **(B)** PINK-siRNA group treated by GM for 24 h showed slightly higher Rhodamine 123 fluorescence but lower DCFH-DA fluorescence compared with NC group. **(C)** PINK1-interfered group showed obviously lesser number of parkin particles in contrast to NC group in response to GM. **(D)** Immunofluorescence staining revealed that PINK1-interfered group showed decreased LC3B (yellow arrows) but more p53 in HEI-OC1 cells (red arrows). **(E)** Total proteins were extracted for agarose gel electrophoresis. Analysis verified that lower PINK1 expression would lead to a decline of LC3B-II, but an increase of p53 and cleaved-caspase 3 by stimulus of GM. **(F)** The results of CCK8 showed that the relative cell viability of PINK1-interfered group declined to some degree in contrast to NC group. Four-hundred micromolar GM exposure could not cause obvious cell death in NC group but in PINK1-silenced group at 24 h. The co-treatment of autophagy inhibitor 3-MA could sharply decline the cell viability both in NC and PINK1-silenced groups. Inhibition of p53 pathway by use of PFTα could increase the cell viability of PINK1-interfered group in response to GM. Results were shown as mean ± SEM, *p < 0.05, **p < 0.01. **(G)** Flow cytometry analysis showed some degree of apoptosis in PINK1-siRNA group treated by GM, which was verified by TUNEL staining.

To further examine the effect of PINK1, we used specific PINK-siRNA to interfere the expression of PINK1 in HEI-OC1 cells. The successful interference of PINK1 led to severer cell damage in morphology (Figure 6A, black arrows) and the decline of cell viability (Figure 6F). We detected more serious mitochondrial membrane potential damage but lower degree of ROS in PINK1-interfered group with GM stimulus for 24 h, reflecting by the stronger fluorescence intensity of Rhodamine 123 and dimmer fluorescence intensity of DCFH-DA (Figure 6B). We treated the NC group and PINK1-knocked down group (PINK-siRNA) with 400 μM GM for 3 h to measure the formation of parkin particles. The red circles of Figure 6C uncovered the reduced degree of parkin particles in PINK1-interfered group in comparison with NC group. In addition, the PINK1-interfered cells showed lower level of LC3B (Figure 6D, yellow arrows; Figure 6E), higher degree of p53 (Figure 6D, red arrows; Figure 6E) and more activated caspase 3 (Figure 6E). Interference of PINK1 expression increased cell death with or without GM exposure (Figure 6F). Of note, autophagy plays a really important role in the protection of cells in response to GM stimulus, since the use of autophagy inhibitor, 3-MA, could efficiently reduce the cell viability under GM stimulus (Figure 6F). On the other hand, inhibition p53 signal pathway by use of PFTα could increase cell viability to some degree in response to GM treatment (Figure 6F). Flow cytometry analysis and TUNEL staining revealed that apoptosis induced by GM occurred in PINK1-silenced group, but not in NC ones (Figure 6G). What’s more, Pearson’s correlation analysis of apoptosis of GM-treated (PINK1-interfered) groups and their corresponding relative expression of PINK1 revealed that GM-induced apoptosis has correlation with the level of PINK1 expression (*P* < 0.05, Supplementary Materials).

## GM Could Influence the Expression of PINK1 in Cochlear Hair Cells of P4 C57BL/6 Mice *in vitro*

**Figure 7:**
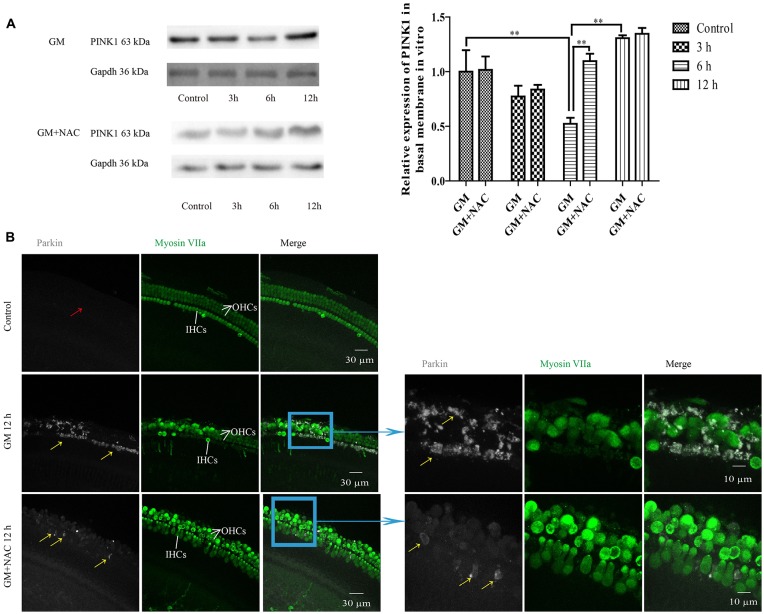
GM could induce the changes of PINK1 expression and formation of parkin particles in hair cells (HCs) of P4 C57BL/6 mice *in vitro*, which could be alleviated by use of NAC. **(A)** GM treatment led to the dynamic changes of PINK1 expression, that is step-by-step decrease after stimulus followed by an increase at 12 h. NAC co-treatment groups showed an instant degradation of PINK1 and a rebound at 6 h and 12 h. **p < 0.01. **(B)** Results showed that 400 μM GM treatment for 12 h could lead to obvious parkin accumulation in HCs (yellow arrows) compared to control group, which showed almost no parkin aggregation (red arrow); and the amount of parkin in HCs could be decreased sharply by NAC co-treatment (yellow arrows).

We then did preliminary research of the function of PINK1 in response to GM treatment in C57BL/6 murine cochlear HCs *in vitro*. As shown in Figure 7A, PINK1 degraded instantly after GM treatment and then rebounded at 12 h. NAC co-treatment alleviated the changes of PINK1 caused by GM.

## GM Could Induce the Activation of PINK1 Pathway, Autophagy and p53 Pathway Related Apoptosis in Cochlear Hair Cells of P4 C57BL/6 Mice *in vitro*, Which Could be Effectively Decreased by NAC Co-treatment

**Figure 8:**
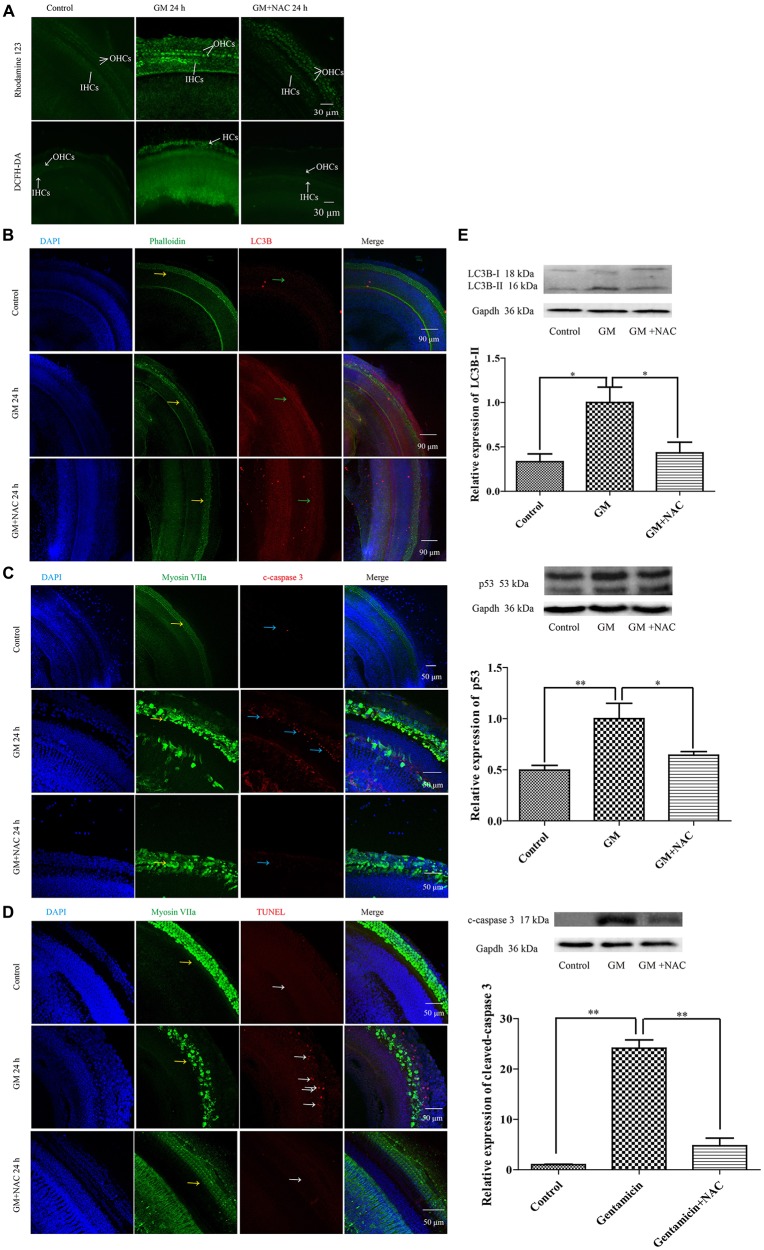
GM could induce the formation of ROS, the damage of mitochondrial membrane potential, an increase of autophagy and some degree of apoptosis in auditory HCs of P4 C57BL/6 mice *in vitro*, which could be effectively decreased by NAC. **(A)** Results of Rhodamine 123 staining revealed the control HCs with dim florescence, GM treated HCs with much stronger fluorescence which could be decreased by use of NAC. The florescence intensity of Rhodamine 123 was in accordance to the DCFH-DA staining, which reflected the level of ROS in tissues. **(B)** GM could cause high level of LC3B (green arrows) and severe HC damages (yellow arrows), which could be decreased by NAC. **(C)** Immunofluorescence staining revealed that there was some activated caspase 3 in HCs after GM treatment for 24 h (blue arrows). ROS scavenger NAC could decrease the level of cleaved-caspase 3 obviously. **(D)** TUNEL staining showed that GM could cause some degree of TUNEL positive nuclei, which was inhibited by NAC (white arrows). **(E)** Western blotting analysis of LC3B-II, p53 and cleaved-caspase 3 revealed the same results with immunofluorescence. Results were shown as mean ± SEM, *p < 0.05, **p < 0.01.

GM treatment for 12 h induced a lot of parkin aggregation in seriously damaged HCs (yellow arrows) in contrast to control group, which could be dramatically reduced by NAC co-treatment (Figure 7B). GM could also lead to an increase of ROS production and mitochondrial membrane potential damage, shown as higher intensity of fluorescence of DCFH-DA and Rhodamine 123 staining in HCs (Figure 8A). Additionally, GM stimulus led to higher levels of LC3B, c-caspase 3 and apoptosis to some degree (Figures 8B–E) in HCs with HCs arranging disorderly, showing abnormal morphology and some of them missing (Figures 8B–D, yellow arrows) compared with control group, the level of which could also be reduced by the co-treatment of NAC. We speculated that ROS was a main damage factor in GM-induced HCs injury, which resulted in HC apoptosis though in which more autophagy was induced trying to protect cells by clearing damaged organelles.

## Discussion

In the current study, we found that exposure of HEI-OC1 cells to 400 μM GM led to the production of ROS in a time-dependent manner, which could be significantly reduced at all the measured time points by use of NAC, as evidenced by DCFH-DA fluorescent staining. This indicates that the accumulation of ROS in HEI-OC1 cells induced by GM may probably be a major cause for GM-related ototoxicity. It has been confirmed that ROS is an effective activator of PINK1/parkin signal pathway in different cell types (Kuznetsov et al., 2017). In this work, data revealed that GM exposure resulted in a sharp decline of PINK1 expression immediately after insult, followed by a gentle increase step by step reaching to the peak at 12 h. The initial degradation of PINK1 suggested the activation of PINK1 pathway induced by GM and, whilst, it is plausible to question the following increase being the result of certain unknown mechanisms driving its expression to the normal level since PINK1 was important for cell maintaining. Interestingly, the expression of PINK1 showed a decline again at 24 h after treatment, which might be attributable to the consistent existence of GM.

On the other hand, NAC co-treatment with GM showed less activity to the changes of PINK1 expression, especially at the early time point. These findings imply that PINK1 is surely influenced by the ROS produced by GM, thereby providing a feasible foundation for our subsequent experiments.

We then detected the distribution of parkin, the main downstream effector regulated by PINK1 (Matsuda et al., 2010), in HEI-OC1 cells in response to the changes of PINK1 expression. Immunofluorescence staining showed that GM exposure contributed to the formation of visible parkin positive particles colocalized with PINK1 and mitochondria in HEI-OC1 cells, which was not observed in control group. Of note, there were some positive parkin particles near but not overlapped with mitochondria, suggesting the initial formation of parkin particles might take place at site adjacent to the damaged mitochondria and, probably, followed by a subsequent translocation to the mitochondria. Notably, the ratio of parkin-particle positive cells went up, reaching peak at 3 h, then, reduced at 6 h followed by a rise at 12 h, which was alleviated but not eliminated totally by use of NAC, indicating that ROS induced by GM treatment was a major but not the only factor accounting for the activation of PINK1/parkin pathway. Additionally, we compared the correlation of PINK1 expression and the ratio of parkin-particle positive cells. Findings showed that the ratio of parkin-particle positive cells changed following the variation of PINK1 expression, as the peaks and the valleys appeared hours later, indicating the activated but delayed downstream function of PINK1.

It is well known that autophagy is responsible for elimination of the damaged organelles within lysosomes, so as to maintain the homeostasis of cells at the early stage under various stresses (Stolz et al., 2014) and one main function of PINK1 pathway is acting as a critical gatekeeper of mitochondrial quality control (Leites and Morais, 2018). So, in the following experiment, we investigated the probable effect of PINK1 on mitophagy under GM stimulus. Results showed the loss of mitochondrial membrane potential and a large number of mitophagy particles appeared in the perinuclear region, a region GM firstly spreading to in HCs (Kalinec et al., 2005). A perfect overlapping of Tom 20 and Lamp-1 was observed in GM treated cells, which was sharply alleviated by co-treatment of NAC, implying that ROS induced by GM could potently trigger serious mitochondrial damage, and cells try to survive through effective degradation of them with the help of lysosomes.

However, to which direction the cells go is dependent on the magnitude of the toxic insult, i.e., if the exogenous stresses exist persistently, which is beyond the self-repaired ability of cells, the ultimate fate of the injured cell is death. Mounting evidence revealed that p53 signal pathway played an important role in GM-induced cell death (Warchol, 2010; Coffin et al., 2013). Our data showed more p53 accumulation and the activation of caspase 3 in GM group compared to control group and NAC co-treatment group, which is an early sign of apoptosis, but without obvious cell death, suggesting that the HEI-OC1 cells exhibited strong resistance to GM-induced cell death.

To verify the correlation between PINK1 pathway and GM-induced cell damage, we further silenced the expression of PINK1 in HEI-OC1 cells. Only PINK1 silence without any additive stresses could induce cell death to some degree, implying that PINK1 pathway might play a role in HC survivals. PINK1-interfered cells showed partially mitochondrial membrane potential loss, fewer parkin particles, lower expression of LC3B, higher level of p53 and c-caspase 3, decreased cell viability and, whilst, lower level of ROS in response to GM stimulus. We speculated that the decrease of ROS production in PINK1-silenced group might be a result of lacking relative healthy mitochondria, since the accumulation of defect mitochondria, which should have been cleared away by PINK1-related mitophagy, might influence the dynamic balance of mitochondrial fission and fusion (Leites and Morais, 2018). In addition, autophagy played a protective role in GM-induced cell damage, since using the autophagy inhibitor, 3-MA, could reduce cell viability both in NC group and PINK1-silenced groups. Moreover, we considered that PINK1 interference made cells more sensitive to the lethal effect of p53 pathway in process of GM insult as evidenced by CCK8 assay. Flow cytometry and TUNEL staining revealed the apoptosis in PINK1-silenced cells in response to GM stimulus. All the data provided proof of concept that PINK1 might protect HEI-OC1 cells from GM-induced cell death via inducing autophagy and inhibiting p53 pathway. Interestingly, the indiscrimination of cell viability between NC groups with and without GM treatment verified the strong resistance of HEI-OC1 cells against GM-induced death.

Lastly, on the basis of experiments in HEI-OC1 cells, we did certain relevant researches in *ex vivo* primary cultured basal membrane of P4 C57BL/6 mice. We found that GM treatment could cause dynamic change of PINK1 expression, parkin aggregation, mitochondrial membrane potential damage, ROS formation, autophagosome induction, p53 and apoptosis up regulation in cochlear HCs, which were mainly associated with ROS induced by GM. The serious cochlear HC death induced by GM indicates the existence of different features between HEI-OC1 cell line and cochlear HCs, which may be due to the loss of numerous supporting cells, repeated cell generations or other unclear factors of HEI-OC1 cells.

Taken together, the findings from the present study reveal that GM is able to activate PINK1/parkin pathway and autophagy mainly by stimulating ROS production, which, in turn, protects HCs from GM-induced ototoxicity via eliminating damaged organelles and inhibiting p53 pathway. We thus argue that PINK1/parkin pathway might be essential for HC survival under GM exposure that may help us fully understand the mechanism of GM-induced ototoxicity.

## Author Contributions

QY mainly responsible for the research and writing process. YZ, HY and HL responsible for the methods. MZ and GS responsible for the software. ZC and RM responsible for the verification. HW and JL responsible for rewriting and fundings.

## Conflict of Interest Statement

The authors declare that the research was conducted in the absence of any commercial or financial relationships that could be construed as a potential conflict of interest.
