# Supplementary material for: PINK1 Protects Against Gentamicin-Induced Sensory Hair Cell Damage: Possible Relation to Induction of Autophagy and Inhibition of p53 Signal Pathway
Source: Front Mol Neurosci. 2018 Nov 12;11:403. doi: 10.3389/fnmol.2018.00403 (PMC6240688; doi:10.3389/fnmol.2018.00403)
Supplement: Supplementary file 1 [file Table_1.doc]

Fig. 1A The ratio of ROS positive cells.

| Paris of group | P value |
| --- | --- |
| Control vs GM (1 h) | 0.039 |
| GM (1 h) vs GM (3 h) | 0.050 |
| GM (3 h) vs GM (6 h) | 0.002 |
| GM (6 h) vs GM (12 h) | 0.000 |
| GM (3 h) vs GM+NAC (3 h) | 0.002 |
| GM (6 h) vs GM+NAC (6 h) | 0.000 |
| GM (12 h) vs GM+NAC (12 h) | 0.000 |

Fig. 1B Mean fluorescence intensity of DCFH-DA

| Paris of group | P value |
| --- | --- |
| Control vs GM (24 h) | 0.005 |
| GM (24 h) vs GM+NAC (24 h) | 0.001 |

Fig. 2 Relative expression of PINK1

| Paris of group | P value |
| --- | --- |
| Control vs GM (1 h) | 0.001 |
| GM (1 h) vs GM (6 h) | 0.003 |
| GM (6 h) vs GM (12 h) | 0.029 |
| GM (12 h) vs GM (24 h) | 0.029 |
| GM (3 h) vs GM (6 h) | 0.025 |
| GM (1 h) vs GM+NAC (1 h) | 0.037 |

Fig. 4A The ratio of parkin-particle positive cells

| Paris of group | P value |
| --- | --- |
| Control vs GM (1 h) | 0.000 |
| GM (1 h) vs GM (3 h) | 0.000 |
| GM (3 h) vs GM (6 h) | 0.002 |
| GM (6 h) vs GM (12 h) | 0.001 |
| GM (1 h) vs GM+NAC (1 h) | 0.005 |
| GM (3 h) vs GM+NAC (3 h) | 0.000 |
| GM (6 h) vs GM+NAC (6 h) | 0.000 |
| GM (12 h) vs GM+NAC (12 h) | 0.001 |

Fig. 5A Rhodamine 123

| Paris of group | P value |
| --- | --- |
| Control vs GM (24 h) | 0.007 |
| GM (24 h) vs GM+NAC (24 h) | 0.028 |

Fig. 5C The number of mitophagy particles per cell

| Paris of group | P value |
| --- | --- |
| Control vs GM (24 h) | 0.000 |
| GM (24 h) vs GM+NAC (24 h) | 0.000 |

Fig.5E LC3B-II

| Paris of group | P value |
| --- | --- |
| Control vs GM (24 h) | 0.001 |
| GM (24 h) vs GM+NAC (24 h) | 0.002 |

Fig.5E p53

| Paris of group | P value |
| --- | --- |
| Control vs GM (24 h) | 0.014 |
| GM (24 h) vs GM+NAC (24 h) | 0.030 |

Fig.5E cleaved-caspase 3

| Paris of group | P value |
| --- | --- |
| Control vs GM (24 h) | 0.001 |
| GM (24 h) vs GM+NAC (24 h) | 0.008 |

Fig.6B GM (NC) vs GM (PINK1-siRNA)

| Paris of group | P value |
| --- | --- |
| Rhodamine 123 | 0.007 |
| DCFH-DA | 0.036 |

Fig.6E GM (NC) vs GM (PINK1-siRNA)

| Paris of group | P value |
| --- | --- |
| LC3B-II | 0.030 |
| p53 | 0.038 |
| c-caspase 3 | 0.031 |

Fig.6F Cell viability

| Paris of group | P value |
| --- | --- |
| NC vs PINK1-siRNA | 0.004 |
| PINK1-siRNA vs GM (PINK1-siRNA) | 0.000 |
| GM (PINK1-siRNA) vs GM+PFTα (PINK1-siRNA) | 0.001 |
| GM (NC) vs GM+3-MA (NC) | 0.000 |
| GM (NC) vs GM (PINK1-siRNA) | 0.000 |
| GM (PINK1-siRNA) vs GM+3-MA (PINK1-siRNA) | 0.000 |
| GM+PFTα (NC) vs GM+PFTα (PINK1-siRNA) | 0.000 |
| GM+3-MA (NC) vs GM+3-MA (PINK1-siRNA) | 0.000 |

Fig.6F Pearson’s correlation analysis

| GM (NC) vs GM (PINK1-siRNA) |  |
| --- | --- |
| Pearson Correlation | 0.772 |
| P value | 0.015 |

Fig.6G Annexin V (gate%)

| Paris of group | P value |
| --- | --- |
| GM (NC) vs GM (PINK1-siRNA) | 0.05 |
| PINK1-siRNA vs GM (PINK1-siRNA) | 0.013 |

Fig. 7A Relative expression of PINK1

| Paris of group | P value |
| --- | --- |
| Control vs GM (6 h) | 0.003 |
| GM (6 h) vs GM (12 h) | 0.000 |
| GM (6 h) vs GM+NAC (6 h) | 0.001 |

Fig.8E LC3B-II

| Paris of group | P value |
| --- | --- |
| Control vs GM (24 h) | 0.012 |
| GM (24 h) vs GM+NAC (24 h) | 0.023 |

Fig.8E p53

| Paris of group | P value |
| --- | --- |
| Control vs GM (24 h) | 0.009 |
| GM (24 h) vs GM+NAC (24 h) | 0.035 |

Fig.8E cleaved-caspase 3

| Paris of group | P value |
| --- | --- |
| Control vs GM (24 h) | 0.000 |
| GM (24 h) vs GM+NAC (24 h) | 0.000 |

Correlation analysis: Apoptosis of GM (PINK1-siRNA) and Relative expression of PINK1 of the corresponding GM (PINK1-siRNA) groups.


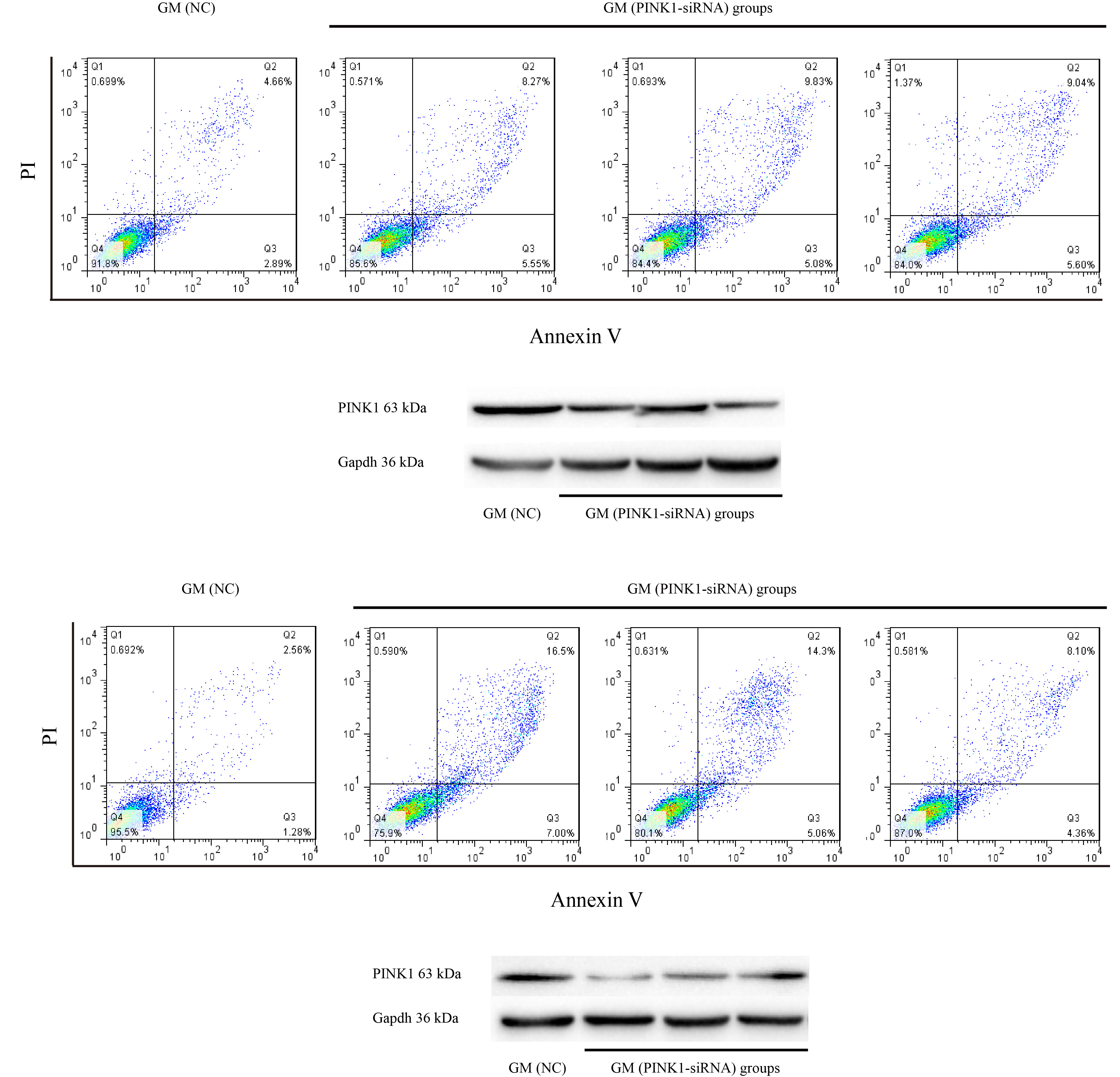


| Pearson Correlation | -0.826 |
| --- | --- |
| P value | 0.043 |
